# Surgery on the Continent of Europe

**Published:** 1887-02

**Authors:** W. S. Caldwell

**Affiliations:** Paris


### FOREIGN CORRESPONDENCE.

### SURGERY ON THE CONTINENT OF EUROPE.

*To the Editor of* THE CHICAGO MEDICAL JOURNAL AND EXAMINER:

Nine years ago I made a tour through Europe, mainly to collect such new ideas as I could obtain at the clinics in the different hospitals.

Lister was at that time still at Edinburgh, and I hurried there to study under the master himself what at that time was considered par excellence antiseptic surgery.

His germ theory of the contamination of surgical wounds was in such perfect accord with the preconceived notions of many German investigators, that I found at both Vienna and Berlin his modes of procedure, with all their paraphernalia, carried out. During a second tour of observation, after the lapse of less than a decennial, I find that the Lister spray is not generally employed, and has, in fact, been banished by the very man who first called it into requisition. After observing the methods of different European surgeons, each claiming that he takes full antiseptic precautions in operating, one is forced to the conclusion that the scientific world does not agree as to what constitutes antiseptic practice in surgery.

Bilroth in Vienna, and Bergmann in Berlin, for instance, take off their coats and vests, put on clean linen operating-gowns, scrub their hands with a brush and soap, and then wash them in a sublimate solution before touching the patient. The surface to be operated upon is washed in the same manner, and shaved with a razor if the parts are covered with an abundant growth of hair. Bergmann is the most painstaking man I have seen operate, and his results are the best; that is, his wounds heal more uniformly without suppuration or constitutional reaction.

He condemns the use of instruments with horn handles, believing that it is difficult to render them aseptic. I had the good fortune to attend his clinic during the college vacation, when he was lecturing to and operating for the instruction of the army surgeons.

There is a feature about his operations that, in my opinion, plays a most important rôle, and that is the care with which he ligates every bleeding vessel, however small. For this purpose he uses only catgut, even to ligate the femoral artery.

For example, I have seen him operate many times for cancer of the breast in the female. He extends his incision from the inner and lower border of the breast as far as the level of the upper border of the axillary space, and thus opens up the entire axilla with one long, continuous incision, from which he not only removes the mamma but the entire chain of axillary glands. By this mode of operating he claims better results than are generally obtained. He claims that the glands in the armpit, although not apparently, are yet very generally affected, and that they should, consequently, be removed. In this operation I have seen him tie as many as sixty blood vessels, and never saw him ligate less than twenty-five. He never uses sponges, but in their stead employs a thin gauze disinfected with the bichloride of mercury. He uses a drainage tube only in cases of large sacks left after an operation. He sprinkles iodoform over the edges of the wound, and covers the same with a thick layer of his gauze. Over this he places antiseptic cotton, and lastly bandages the parts firmly. If the patient has no constitutional reaction in the way of fever, he leaves this first dressing in place from three to five days. Erysipelas is an accident which he never seemed to fear, and I never saw a case in his wards.

---

Hospital Necker, Paris. Service of Professor Le Fort. November 12th, 1886.—The first case was a woman, 30 years of age, an employe in a match factory, suffering from a disease of the inferior maxilla, caused by phosphorus poisoning. Professor Le Fort removed without much difficulty a large sequestrum, scooped out the cavity with a sharp spoon, and then plugged it with iodoform gauze.

The next patient was a woman of 60 years of age, from whom Dr. Le Fort had removed the breast for cancer some years before, which had now reappeared in the old cicatrix He spoke, before beginning to operate, of the danger of erysipelas after operations of this kind in women of this age, and of the necessity of using all necessary antiseptic precautions to prevent its occurrence; but he proceeded to operate after simply washing his hands in soap and water, using no brush and not cleaning his finger nails. He did not tie a single artery, using torsion to arrest the bleeding, and applied the thermo-cautery to such vessels as still bled after this procedure.

The third case was that of a workman, 23 years of age, who some months previously had severed the extensor tendon of the thumb at the middle of the metacarpal bone. The operation consisted in reopening the wound and uniting the severed ends of the tendon. Dr. Le Fort said that formerly such operations had failed, but that since the introduction of antiseptic surgery better results had been obtained. The patient was a little slow in pulling off his old dirty shoes after he had mounted upon the operating table, and Dr. Le Fort untied and pulled off the last shoe himself, and then proceeded to operate without washing or cleaning his hands in any way. The manner of uniting the two ends of the tendon was instructive. He used catgut and passed the needle through one tendon, tied his ligature so as to ligate about one third of its circumference, and then passed the needle through the other end of the tendon and tied that in the same manner. By this means the ligature was firmly attached to both ends of the tendon. He claimed that if one simply sutured the parts together as one would the edges of a wound in the skin, the direction of the fibers was such that the suture would not hold.

Professor Galezowski, whose eye clinic I consider one of the very best in Europe, is always talking about antisepsis. After his cataract operations he puts into the eye a gelatin disc impregnated with the bichloride of mercury, and then puts over the eye a compress of iodoform gauze. In his last 600 operations he has only had sloughing of the cornea in three cases. Yet he will examine and treat from twenty-five to fifty cases of disease of the external eye of every imaginable form, and proceed to operate after simply wiping his fingers on a towel wet in a solution of carbolic acid Nothing suits a Frenchman better than an opportunity to hold a German up as an object of ridicule. Dr. Galezowski took occasion to gratify this taste one day by giving us a synopsis of the rules laid down at a late meeting of the Ophthalmological Society of Hamburg.

HOPITAL DE LA PITIÉ.

At this hospital I saw the most bungling laparotomy I ever have seen performed, excepting, perhaps, one ten years ago, performed by a gynæcologist who at that time, and even to-day, is recognized as one of the first authorities in England.

To see what a perfect failure two such great authors on the subject of diseases of women are, when they attempt to do an ovariotomy, has led me to the conclusion that there is an especial skill required for the performance of this operation which a few men possess, and many cannot acquire. This man's idea of antisepsis was evidently the use of the spray, for while the room was so full of the vapor of carbolic acid that one could hardly breathe, one of his assistants had just finished dressing a woman's leg affected with acute phlegmasia dolens, and went directly from that case to the operating room without changing his clothing. The man who handed the instruments had dirt under his finger nails, and the numerous assistants who attended to the sponges, did not wash their hands after coming into the operating room. The cyst was one of the broad ligament, it had a great many attachments, and the operation was a difficult one. The patient died twenty-four hours after the operation, but whether from carbolic-acid poisoning or from the operation itself I was unable to settle in my own mind.

I attended A. Martin's vacation course for physicians at Berlin, and will give a brief résumé of his idea of antisepsis.

The room where he operates is impregnated the evening before the operation with a ten per centum spray of carbolic acid, and then closed up tightly for the night. In the morning before the operation, he, his two assistants and the nurse, each take a bath and put on entirely clean clothing. Every one of his class who is to witness the operation is required to take a full bath just previous to entering the room as well as to change his clothing throughout.

Those who touch his instruments, that is, his nurse and one assistant, are required to wash their hands thoroughly in a solution of bichloride of mercury.

Martin operates with a rapidity that is wonderful. I have seen him remove an ovarian cyst and close the wound in seven minutes, and have frequently seen him do the operation inside of ten minutes. He considers dispatch a prime element of success, and thinks that Lawson Tait's idea of a small abdominal incision is an erroneous one.

He thinks that too many assistants are bad and tells of performing an ovariotomy entirely unassisted, those he depended upon being taken with a fainting fit and hence unable to perform their duty.

Professor Verneuil still continues to teach at La Pitié and is as full of vigor and energy as he was when I first heard him twenty years ago. My time has been so much occupied in other directions that I have not been able to follow him regularly. He has, however, a hobby which he is riding just now, and that is, the discussion of glandular tumors by the interstitial injection of a ten per centum solution of iodoform in ether. His results are apparently very good.

Hôpital Saint Louis contains 1,043 beds, 625 of which are devoted to diseases of the skin. Under the management and teaching of Professor Fournier, it is one of the best places in Europe to study this branch of medicine.

M. Péan is the surgeon in chief of this hospital, where more than 2,000 cases of a surgical character are annually treated. He is a bold and rapid operator and a fine teacher. He talks as every one else does of antisepsis, although he wears each day the same old black swallow-tailed coat during his operations, upon which the accumulation of blood-spots date back many months. He always washes his hands between each operation in soap suds, but not carefully, from a German standpoint. In his ovariotomies he is more careful, disinfecting his hands, sponges and instruments with the bichloride of mercury.

Among the first patients I saw Pean operate on this winter was a man 36 years of age, who had an attack of pleurisy two years before. The pleural cavity on the left side secreted a large amount of fluid, which was removed two or three times by the aspirator.

Some eight months ago a complication occurred which Pean remarked was very unusual, and that was an artificial opening was established between the ninth and tenth ribs on a line with the nipple, from which pus flowed nearly constantly, but not sufficiently to keep the pus-sac empty. To establish perfect drainage, Péan resected a portion of the seventh, eighth and ninth ribs, and when he had thus laid bare the pleural sac, he opened the same widely with the thermo-cautery, and evacuated three litres of pus.

About the time the pus ceased to flow the patient ceased to breathe and died on the table in spite of every effort to resuscitate him. Pean attributed the death of this patient, not to the anæsthetic, but to the sudden movement of the heart toward its natural position from which it had been displaced toward the right by the fluid. This is the only place where I have seen used the apparatus of the late lamented Paul Bert for the administration of chloroform. Its inventor claimed that by its use atmospheric air and the vapor of chloroform were mixed in such a proportion, always definite and the same, as to render the use of this most valuable anæsthetic perfectly and absolutely safe. The instrument is an expensive and complicated one and has not been generally adopted even here in Paris.

This is the fourth patient I have seen die on the operating table under chloroform since I came to Europe. Of this number one occurred in the service of an interne at Vienna, in the operating room of Professor Albert.

The patient was a young woman 23 years of age, apparently in perfect health, and was being operated upon for the removal of a lymphoma the size of a small hen's egg situated at the outer angle of the inferior maxillary bone.

Not more than two or three drachms of chloroform had been given when the patient ceased to breathe, and no amount of artificial respiration would resuscitate her. A careful post-mortem failed to give any explanation of the cause of death, as the heart was normal and no air had gained access to the circulation.

I saw two other patients come near dying, one being operated on by Schroeder and the other by Bergmann.

Shroeder, by the way, always has a battery at hand and ready for use in such an emergency.

In Bergmann's case he executed a little maneuver that he places great stress upon; and that is, while his assistants execute rapidly the movements necessary for artificial respiration he introduces his finger into the mouth and holds up the the epiglotis. He claims that this organ will often close down firmly and that manipulations are of no use unless one operates in this manner.

December 11th, 1886. The first patient to-day was a woman 33 years of age, married, but had never borne children.

She had suffered severely from dysmenorrhœa since the first appearance of her menses at the age of eighteen. Lately she had not only suffered at her menstrual periods, but was confined to the bed for a large part of every month with excruciating pains through her pelvis, mostly on each side running in the direction of the broad ligaments.

Frequent attacks of an hysterical character accompanied these symptoms.

Physical examination failed to indicate any pathological change on the part of the pelvic organs. Péan is fond of talking, and this patient furnished him a text for a long discourse on the subject of the castration of the female for nervous disturbances as practiced by Hegar, Battey and others.

He said that he had practiced the removal of the ovaries frequently in this class of cases, but had usually found the proceeding of little or no benefit. He said that the relation which the tubes and ovaries bear to these manifold nervous manifestations might be problematic, but that they sustained an intimate relation to the womb itself, was an unquestionable fact. Therefore he thinks that the more rational practice is the total extirpation of the womb.

This operation he proceeded to perform in a manner different from that practiced by Karl Braun and Martin.

Beginning at the posterior vagino-cervical junction, keeping close to the uterine tissue, he cut with the scissors until he had opened into the peritoneal cavity through the cul-de-sac of Douglas.

To prevent and arrest hæmorrhage he applied his hæmostatic forceps in such a manner as to completely include the free margin of the incision he had made.

He now began the same dissection anteriorly separating the uterus from the bladder until he had opened into the peritoneal cavity.

He next introduced the index finger into the lower opening and brought it out at the upper one, and thus held the broad ligament of one side within his grasp. This he clamped by passing a long-bladed hæmostatic forceps from above downward and another from below upward, and thus having all the tissues of this membrane compressed, he divided the same close to the womb. Each broad ligament was treated in the same manner.

When the operation was completed, there were eleven pairs of forceps projecting from the vagina which he said he should leave in place, the smaller ones 24 hours, and the larger ones 48 hours. The vaginal cavity was washed out with a bichloride solution, tamponed with iodoform gauze, and the patient put to bed. Very little blood was lost.

The womb was cut open and showed only a slight endometritis of the body. I took pains to look after this patient, as to the outcome, and as far as the operation was concerned she made a rapid recovery with very little constitutional disturbance, but as to the relief of the symptoms for which this desperate operation was done enough time has not yet elapsed to determine that most important question.

During the last meeting of the North-German Society of Physicians at Berlin I attended pretty regularly the section devoted to gynæcology.

The subject of the removal of the ovaries for the relief of nervous disturbances in women was freely discussed at one of these meetings.

Hegar, Schrofyder, Veit, Sanger, Gusserow and others took part in the discussion and the sentiments expressed were generally of the most conservative character. The conclusion was general that the operation in the past had been too frequently performed; that healthy ovaries ought not to be removed, and that the operation was unjustifiable in cases where the nervous phenomena produced little or no effect upon the general health of the patient. In fact the operation should be limited to those cases where life is in jeopardy.

December 18th, 1886. The first case before us to-day was a girl, 17 years of age, brought here from one of the provinces. Eight years ago her parents noticed a small tumor just beneath the right ear. It increased in size until at the present date it is seven inches long and four inches wide and firmly attached, and seemed to take its origin from the side of the cervical vertebra.

Pean remarked before beginning to operate that this was one of the most difficult operations in surgery, as the tumor involved some of the most important organs in the body. He says never attempt to enucleate a tumor like this situated in this position. Open it up from the surface and cut straight through the mass from above downward until the base is reached. This can be done without hesitation as one never finds important vessels or nerves in the body of the tumor itself. When the bottom of the mass is reached one may find it loose and easily detached. If it is firmly adherent to important tissues, cut it away piece-meal as far as possible, and leave the balance to slough away, which it will do after being isolated from the surrounding parts. This formidable mass, however, he succeeded in removing entirely.

OPERATION FOR STONE.

Pean, like Bergmann and Guyon, performs the supra-pubic section. Every teacher and operator I have seen in Europe discards the perineal section. Crushing small calculi and removing larger ones by the high operation seems to be almost a universal rule at the present time.

Professor Lannelogne in his lectures at the School of Medicine treated, for the first part of the session, of injuries and diseases of the head, spine and neck.

For injuries of the skull he still adheres mainly to the use of the trephine, claiming that when the operation is performed antiseptically it gives good results. On the other hand Bergmann has abandoned this instrument, and in a late number of Volkmann's Vorträge he takes occasion to discuss this subject in a most thorough manner, showing that by substituting the hammer and chisel, immensely better results are obtained.

In speaking of Pott's disease of the spine Professor Lannelogne says that the microscope has settled the question as to its tuberculous origin.

As to the plaster-of-Paris jacket of Sayre, he says that the idea of keeping the two diseased surfaces separated by this appliance is irrational and impracticable. He puts his patient in a horizontal position and keeps him there for months together, taking him in the open air as much as possible to preserve the general health. He says when one has a psoas abscess as the result of this disease never aspirate it. Open it up freely, curette carefully as much of the sac as one can, and then inject with iodoform in weak solution to cleanse the puscavity. I have seen, by the way, a long-handled wooden spoon used for this purpose.

The Hôpital des Enfants Malades contains 600 beds. M. de Saint Germain is the surgeon-in-chief to this institution. In his rough manner of operating and dealing with the poor unfortunates who are under his charge he puts one in mind of the model surgeon of a century ago. He always chloroforms his patient himself. In doing this he pours about three drachms of chloroform on a towel, three or four double, claps it over the face of the child, and, despite its cries and struggles, allows it no air except what it can get through the meshes of this cloth. His patient is completely insensible in one minute. He claims this to be the safer mode of giving chlorform, as less of the drug is required to produce anæsthesia. His surgery, like his administration of chloroform, is rapid and rough. Chronic enlargement of the tonsils he treats with the thermo-cautery. He uses a small point which has a direction of a right angle to the main handle, and this he introduces at two points into the tumor. Usually two sittings cure the patient. In older children, where the tonsil has become much indurated, he incises the organ with the knife.

The tonsillitome he says is an instrument designed for a physician but not for a surgeon. In the treatment of large nævi, after injecting the center of the mass with the solution of iron, he destroys the small points that are generally left around the margin with the actual cautery. He has had, he says, two deaths occur in his practice from the injection of the iron solution, and since then practices great care in the use of a ring around the border of the mass which he presses down firmly during the operation and for a little time afterwards.

Phimosis he treats by dilating the prepuce instead of by circumcision.

For this operation he uses a pair of pointed forceps which he introdces as far as the meatus and then spreads it until he can pass the gland through the opening. The operation is followed by less irritation than circumcision and the results seem to be good.

He is not a friend of the Jewish idea of the superfluity of the foreskin; in fact, he says that when you cut it away you have in a manner deformed the patient and deprived him of a tissue which he believes to contribute to the perfect healthiness of this most important gland. W. S. CALDWELL.

PARIS, December 28th, 1886.

